# Infusion of Bone Marrow Mesenchymal Stem Cells Attenuates Experimental Severe Acute Pancreatitis in Rats

**DOI:** 10.1155/2016/7174319

**Published:** 2016-09-18

**Authors:** Hang Zhao, Zhiying He, Dandan Huang, Jun Gao, Yanfang Gong, Hongyu Wu, Aifang Xu, Xiangjun Meng, Zhaoshen Li

**Affiliations:** ^1^Department of Gastroenterology, Shanghai First People's Hospital, School of Medicine, Shanghai Jiao Tong University, Shanghai 200080, China; ^2^Department of Cell Biology, Second Military Medical University, Shanghai 200433, China; ^3^Department of Emergency, Changhai Hospital, Second Military Medical University, Shanghai 200433, China; ^4^Department of Gastroenterology, Changhai Hospital, Second Military Medical University, Shanghai 200433, China; ^5^Department of Gastroenterology, Shanghai Sixth People's Hospital, School of Medicine, Shanghai Jiao Tong University, Shanghai 201306, China

## Abstract

*Background & Aims*. Severe acute pancreatitis (SAP) remains a high-mortality disease. Bone marrow (BM) mesenchymal stem cells (MSCs) have been demonstrated to have plasticity of transdifferentiation and to have immunomodulatory functions. In the present study, we assessed the roles of MSCs in SAP and the therapeutic effects of MSC on SAP after transplantation.* Methods*. A pancreatitis rat model was induced by the injection of taurocholic acid (TCA) into the pancreatic duct. After isolation and characterization of MSC from BM, MSC transplantation was conducted 24 hrs after SAP induction by tail vein injection. The survival rate was observed and MSCs were traced after transplantation. The expression of TNF-*α* and IL-1*β* mRNA in the transplantation group was also analyzed.* Results*. The survival rate of the transplantation group was significantly higher compared to the control group (*p* < 0.05). Infused MSCs were detected in the pancreas and BM 3 days after transplantation. The expression of TNF-*α* and IL-1*β* mRNA in the transplantation group was significantly lower than in the control group in both the pancreas and the lungs (*p* < 0.05).* Conclusions*. MSC transplantation could improve the prognosis of SAP rats. Engrafted MSCs have the capacity of homing, migration, and planting during the treatment of SAP.

## 1. Introduction

Acute pancreatitis comprises a mild, self-limiting disease and in about one-third of patients an inflammatory process that causes local and systemic complications, frequently resulting in a systemic organ dysfunction. Despite improvements in diagnostics and therapeutics, the mortality rate of severe acute pancreatitis (SAP) has remained high over the past decade. About 5–10% of patients develop necrosis of the pancreatic parenchyma, and patients with peripancreatic necrosis alone have increased morbidity rates compared to patients with interstitial edematous pancreatitis [1]. Fulminant pancreatitis and subfulminant pancreatitis have a mortality rate greater than 70% [2]. Systemic inflammatory response syndrome (SIRS) plays an important role in the development of multiple organ dysfunction syndrome (MODS), which is the primary cause of morbidity and mortality. Treatment of acute pancreatitis includes nutritional support, use of prophylactic antibiotics, and fluid replacement, but until now there is no effective therapy for SIRS. The prevention and blockade of SIRS have been attempted experimentally at different levels and with diverse outcomes. Experimentally the genetic deletion of TNFR-1, the inhibition of TNF-*α* production, and the administration of anti-TNF-*α* have been shown to reduce tissue damage and improve the survival rate of animal models [3–5]. However, the neutralization of TNF-*α* has a very modest impact on mortality in human sepsis [6]. Paradoxical results have been observed for other cytokines, such as IL-1*β*, PGE2, and MCP-1 [7–9]. Nuclear factor *κ*B (NF-*κ*B) signaling pathway also involves the inhibition of SIRS. Many researchers propose that blocking NF-*κ*B activation is beneficial in the treatment of acute experimental pancreatitis [10–13], but the opposite effect is recently reported [14]. The truncation of NF-*κ*B results in severe necrotizing pancreatitis associated with lung inflammation and liver damage [15]. A previous investigation has shown that inhibiting NF-*κ*B increases apoptosis and necrosis [14]. In addition to the difficulty in the inhibition of SIRS, pancreatic necrosis is another challenging problem in pancreatitis treatment, which often leads to infection and increases the risk of the repeated development of SIRS and MODS [16]. No measures are currently available to attenuate necrosis and promote regeneration.

Stem cell biology is becoming a field of interest for researchers from different disciplines. The differentiation ability of tissue-specific stem cells was once thought to be confined to the tissue of origin; however, recent studies suggested that these cells can also differentiate into other lineages [17–20]. Bone marrow (BM) mesenchymal stem cells (MSCs) are a subpopulation of undifferentiated cells in the BM that can differentiate into bone and cartilage, tendon, muscle, fat, and liver tissue. Of particular interest, intravenously infused MSCs specifically migrate to sites of injury. The ability of implanted MSCs to seek out the site of tissue damage has been demonstrated in bone or cartilage fracture, myocardial infarction, pancreatic islets [17–21], and ischemic cerebral injury [22]. Locally distributed MSCs have been reported to differentiate into gastrointestinal epithelial cells or cardiomyocytes after myocardial infarction (MI) [23]. Thus, the transplantation of BM stem cells is considered an alternative treatment for promoting organism regeneration. Engrafted MSCs have been previously proven to promote nerve growth and ameliorate functional deficits after cerebral artery occlusion [24]. The precise mechanisms of regeneration after the transplantation of MSCs are unknown. The immunomodulatory functions of MSCs may also be involved in the restoration process. MSCs can produce soluble factors, modulate many T cell functions, and inhibit the IL-2-induced proliferation of natural killer cells [25, 26]. Jung et al. have shown that administration of the human MSCs alleviated SAP in rats [27]. In another study, L-arginine induced SAP was relieved by mobilization of self-bone marrow MSCs in mice [28].

In our study, we created the induced experimental SAP on SD rats and isolated MSCs from the same rat strain. We hypothesize that acute pancreatitis as a systematic inflammatory disease could be alleviated by MSC transplantation, and the regeneration of pancreatic necrotic tissue may occur following the targeted migration of MSCs into injured regions.

## 2. Materials and Methods

### 2.1. Animal Models

All animal studies were conducted following approval by the Ethics Committee for the Use of Experimental Animals of the Second Military Medical University. The animals used in this study received humane care in compliance with the Guide to Care and Uses of Experimental Animals formulated by the Medical Ethical Committee on animal experiments of the Second Military Medical University. Male or female SD rats weighting 200–250 g were maintained at 23°C with a 12 h light-dark cycle with free access to water and standard laboratory chow. Beginning 24 hrs before the start of the experiment, the rats were deprived of food but were allowed free access to water. The animals were randomized into different groups according to the aim of the study, and a control group was established in each experiment.

### 2.2. Induction of Pancreatitis

Experimental SAP was induced by the injection of taurocholic acid (TCA) (Sigma, St. Louis, MO, USA). This method has previously been described on a mouse model [29]. We injected 3% TCA into the biliopancreatic duct at a rate of 12 mL/hr using a microinfusion pump. About 0.5 mL liquid was injected into the pancreatic duct of each rat. Methylene blue was routinely included in the infusion solution to ensure the success of intubation and exclude animals in which the infusion had extravasated from the duct. The entire infusion procedure, from anesthesia induction to the closure of the laparotomy incision, was generally completed within 20 minutes. The controls were injected with the same volume of saline in the pancreatic duct.

### 2.3. Evaluation of Pancreatitis Severity

To evaluate the severity of pancreatitis and to choose an appropriate treatment time point, rats were euthanized at 0.5 hr, 2 hrs, 4 hrs, 6 hrs, 8 hrs, 12 hrs, and 24 hr after SAP induction according to grouping. We also induced mild acute pancreatitis (MAP) model by a total of four intraperitoneal injections of cerulein (20 *μ*g/kg) with 1 hr intervals, as previously described and validated in our laboratory. Blood samples were obtained from the inferior vena cava and then centrifuged at 4°C. Plasma was collected and stored at −80°C for further measurement of plasma amylase activity, alanine aminotransferase (AAT), and serum creatinine (SCr) levels (Sigma, St. Louis, MO, USA). Pancreatic tissues located within 1 cm of lesser duodenal curvature and inferior lobe of the left lung tissues were removed and cut into small blocks for further studies, including H&E staining, an immunohistochemical study, and RT-PCR detection of TNF-*α* and IL-1*β*. Tissue damage in the pancreas was quantitatively measured with Schmidt's histopathologic scoring criteria [30].

### 2.4. Isolation and Culture of MSCs

BM samples were collected from the femurs and tibias of healthy adult donor rats. The washed cells were resuspended in MSC medium, consisting of Dulbecco's modified Eagle's medium (DMEM) with low glucose supplemented with 10% fetal bovine serum (FBS), 100 U/mL penicillin, 100 mg/mL streptomycin, and 2 mmol/L L-glutamine. The cells were plated at 5 × 10^5^ cells/100 mm dish. Cultures were maintained at 37°C in a humidified atmosphere with 5% CO_2_. After 24 hr, nonadherent cells were removed by changing the medium, and the plate was washed twice with PBS. The medium was replaced every 3-4 days thereafter. When the cultures were near confluence, the cells were detached with 0.05% trypsin/25 mmol/L EDTA solution and replated at a density of 2 × 10^5^ cells/75 cm^2^ flask.

### 2.5. Immunophenotyping of MSCs

Surface markers were selected according to a previous report [31]. The cells were harvested by treatment with 0.05% trypsin-EDTA at the end of the third passage. For flow cytometry, the detached cells were washed and resuspended in PBS. Aliquots of 2 × 10^5^ cells were labeled with fluorescein isothiocyanate (FITC) (BioLegend, San Diego, USA), phycoerythrin (PE) (BioLegend), and PE/CY5-conjugated monoclonal antibodies including an isotype control, anti-CD44-FITC (AbDSerotec, Oxford, UK), anti-CD29-PE/CY5, and anti-CD45-PE (BioLegend). Flow cytometry was performed with a FACSCalibur flow cytometer (Becton Dickinson, USA). In total, 10,000 events per sample were collected into listmode files and analyzed using WinMDI 2.9 analysis software.

### 2.6. Differentiation Assay

The differentiation capacity along adipogenic, osteogenic, and chondrogenic lineages is the golden criterion to identify characterization of MSCs. In our study the capacity of MSCs to differentiate along adipogenic and osteogenic lineages was assessed as previously reported [32]. Briefly, confluent MSC cultures at the end of the third passage were induced into adipogenic differentiation by culturing in adipogenic induction medium containing 1 *μ*mol/l dexamethasone, 0.5 mmol/L 3-isobutyl-1-methylxanthine (IBMX) (Sigma), 10 *μ*g/mL insulin, 200 *μ*mol/l indomethacin (MDI + I) (Sigma), and 10% FBS in DMEM for 48 hr. The medium was then changed to adipogenic maintenance medium containing insulin (10 *μ*g/mL) and 10% FBS in DMEM for 5 days. The cells were stained with 1-8-(4-dimethylphenylazo)-2-naphthalenol (Oil Red O). To induce osteogenic differentiation of the MSCs, the cells were treated with medium consisting of DMEM, 10% FBS, 50 *μ*mol/l ascorbic acid, 10 mmol/L *β*-glycerol phosphate, and 100 nmol/l dexamethasone (Sigma). The medium was replaced every 3 days. Calcium deposition was examined by Alizarin Red S staining 14 days later.

### 2.7. MSC Transplantation

SAP was induced in 8-to-10-week-old female SD rats by TCA injection into the biliopancreatic duct as described earlier. The animals were then randomly divided into a treatment group and a control group (*n* ≥ 10). MSCs from male donor rats were infused into the tail veins of the treatment group at a dose of 5–7 × 10^7^ cells per rat 24 hrs after SAP induction. The control group rats were injected with the same volume of PBS. The rats of both groups that survived were sacrificed 72 hrs after transplantation, and blood and tissue samples were obtained to evaluate the severity of pancreatitis and to investigate the migration and differentiation of the MSCs. The survival rates of the remaining rats were recorded as described previously.

### 2.8. Migration Assay

The migration of MSCs was determined by PCR detection of male-derived MSCs in tissues. Genomic DNA was prepared from the tissues of multiple organs from the transplantation and control groups by the traditional phenol-chloroform method. The sex-determining region of the Y chromosome (SRY) was detected by PCR. Primer sequences for the SRY gene (forward 5′-CATCGAAGGGTTAAAGTGCCA-3′, reverse 5′-ATAGTGTGTAGGTTGTTGTCC-3′) and PCR conditions have been previously published [33]. The amplification product was 104 bp in length. The PCR products were separated using 2% agarose gel electrophoresis and stained with ethidium bromide. Positive (male SD rat genomic DNA) and negative (female SD rat genomic DNA) controls were included in each assay.

### 2.9. Assay for Cytokine Expression

Cytokines expression in the lung and pancreas with and without MSCs transplantation was determined by Real-Time Quantitative PCR (qRT-PCR) method. Total RNA was extracted using a Ferment kit according to the manufacturer's recommendations (Ferments). cDNA was generated from 5 *μ*g of total RNA using oligo dT primers and superscript II reverse transcriptase (Invitrogen), and qRT-PCR was performed using primers specific for the cytokines of interest. Samples were analyzed with the ABI 7500 Sequence Detection System (Applied Biosystems, Foster City, CA). Quantification of the product was measured by the amount of cDNA amplified and using amplification reactions of a 200 bp fragment from *β*-actin cDNA as control. The amount was normalized using *β*-actin as a standard. The primers used were IL-1*β* (230 bp), 5′-ATG AGA GCA TCC AGC TTC AAA TC-3′ and 5′-CAC ACT AGC AGG TCG TCA TCA TC-3′; TNF-*α* (211 bp), 5′-ACT CCC AGA AAA GCA AGC AA-3′ and 5′-CGA GCA GGA ATG AGA AGA GG-3′; *β*-actinin (211 bp), 5′-CCT GTA CGC CAA CAC AGT GC-3′ and 5′-ATA CTC CTG CTT GCT GAT CC-3′. PCR products were also electrophoresed on 2% agarose gels containing 0.1 *μ*g/mL ethidium bromide and photographed under UV transillumination.

### 2.10. Statistical Analysis

All data are presented as the mean ± SD. Continuous variables were compared using Student's *t* test and the Mann-Whitney *U* test, and multiple comparisons were performed using the Kruskal-Wallis test and ANOVA with a Bonferroni correction using SPSS 13.0. *p* < 0.05 was considered significant.

## 3. Results

### 3.1. Characterization of Experimental Acute Pancreatitis

**Figure 1:**
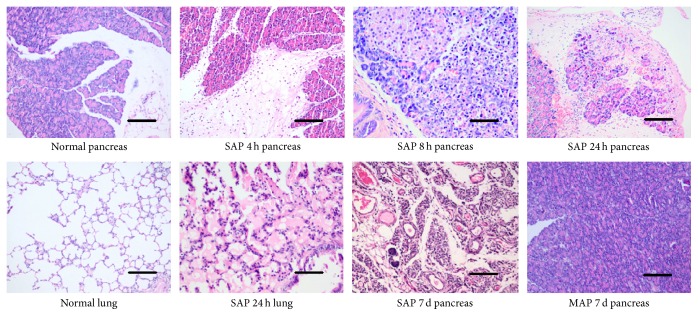
Histological findings of pancreatic and pulmonary tissues after AP. The scale bars are equal to 100 *μ*m in all of the images. (Normal pancreas) pancreatic acini of a control animal; the glandular architecture was entirely normal (H&E, ×200). (SAP 4 h pancreas) pancreatic interstitial edema and infiltration of a great deal of inflammatory cells 4 h after SAP (H&E, ×200). (SAP 8 h pancreas) necrosis of pancreatic acini and hemorrhage in parenchyma 8 h after SAP (H&E, ×200). (SAP 24 h pancreas) severe necrosis, hemorrhage, and infiltration of inflammatory cells 24 h after SAP; structure of pancreatic acini was damaged obviously (H&E, ×200). (Normal lung) pulmonary alveoli of a control animal (H&E, ×200). (SAP 24 h lung) interstitial edema and effusion in alveolar space 24 h after SAP (H&E, ×200). (SAP 7 d pancreas) seven days after SAP induction; pancreatic acini were replaced by granulation tissue and there was significant proliferation of condulets. Tubular complex formation could be found (H&E, ×200). (MAP 7 d pancreas) seven days after mild acute pancreatitis induction, pancreatic tissue almost recovered and no obvious necrosis or infiltration could be found (MAP was induced by repeated cerulein intraperitoneal injection in a previous study).

After SAP induction, the rats showed signs of illness, such as piloerection and extrados, and were in low spirits after pancreatitis induction. Ascites developed in nearly all SAP rats, with diverse volumes and characteristics. Serum amylase activity was significantly elevated 0.5 hr after TCA injection and reached a peak value at 24 hrs that was significantly higher compared to the control group (*p* < 0.05). Serum AAT levels were significantly increased at 8 hr after SAP, and serum creatinine levels were significantly increased at 24 hrs after SAP (*p* < 0.05). At 24 hrs after SAP induction, the acinar architecture was partially destroyed, with focal acinar cell necrosis, interstitial edema, and inflammatory infiltrate, and tissue damage was greater than at 12 hrs. We choose 24 hrs after SAP induction as the treatment timepoint (Figure 1(a) and Table 1).

### 3.2. Characterization of BM MSCs

MSCs were successfully cultured and expanded. The average time required for MSC adhesion was 24–72 hrs. The adherent cells were typically triangular and fusiform in shape. The MSCs tended to grow into colonies. Symmetric colonies were observed at approximately 5–7 days after initial plating. Primary cultured BM cells contained heterogeneous cell populations, among which MSCs were no more than 50%. After serial passage, a homogenous population of fibroblast-like cells was observed.

**Figure 2:**
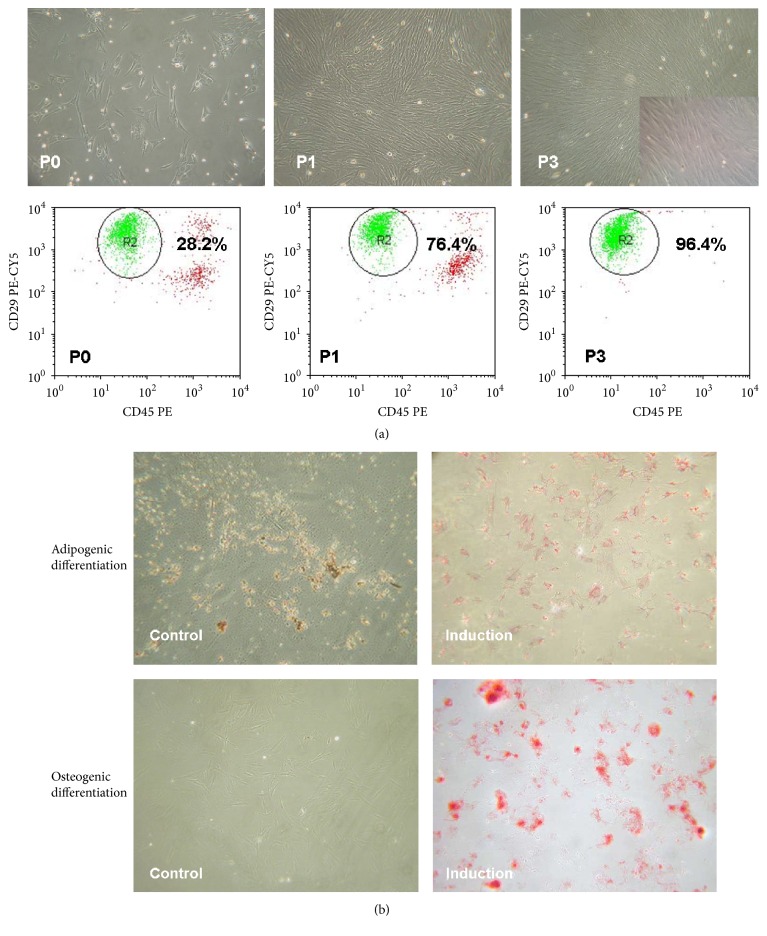
(a) Typical cell shapes in primary culture, the adherent MSCs were triangular and fusiform and other cell shapes were also present (×200). P1 MSCs showed a fibroblast-like shape and whirlpool-like distribution (×100). Whirlpool-like distribution of the third passage of MSCs (×100). Embedded image shows P3 MSCs observed under phase contrast microscopy (×400). Flow cytometric analyses of CD29^+^CD44^+^CD45^−^ cells in MSCs at P0, P1, and P3; the percentage of Lin-CD29^+^CD44^+^CD45^−^ cells gated in R2 was 28.2%, 76.4%, and 96.4%, respectively. (b) Negative Oil Red O staining of control group MSCs at P3 MSCs were cultured in common medium before staining (×100). Positive Oil Red O staining of MSCs at P3 after 5 days of adipose differentiation induction; fat drops in MSCs are stained red (×100) in Negative Alizarin Red S staining of control group MSCs at P3; MSCs were cultured in common medium before staining (×100). Positive Alizarin Red S staining of MSCs at P3 after 14 days of osteocyte differentiation induction, calcium deposition in MSCs stained red (×100).

The homogeneity of the primary passage, passage 1, and passage 3 MSCs was evaluated by a flow cytometric analysis of expressed antigens. Gated MSCs were uniformly positive for CD29 and CD44 but negative for CD45, which was consistent with the MSC phenotype and excluded contamination by hematopoietic cells. The percentage of Lin-CD29^+^CD44^+^CD45^−^ cells at passages 1 and 3 was significantly higher compared to the primary passage (*p* < 0.05) (Figure 2(a)).

The differentiation capacity of the MSCs was also determined. The cells at passage 3 readily differentiated into Oil Red O-positive adipocytes or Alizarin Red S-positive mineralizing cells when incubated in the respective differentiation media. The cells incubated without any differentiation stimuli were negative for both Oil Red O and Alizarin Red S, suggesting that these cells were not contaminated with either adipocytes or osteocytes, respectively (Figure 2(b)).

### 3.3. Effects of MSC Transplantation on SAP

**Figure 3:**
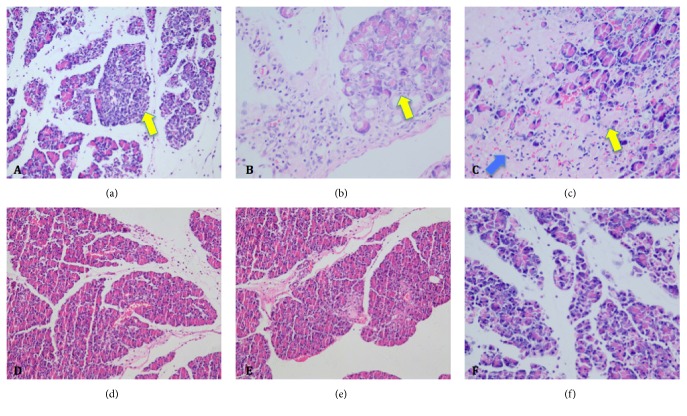
(a–c) Histological findings of pancreatic tissues 72 hrs after PBS infusion (H&E; (a) ×200; (b, c) ×400); (d-e) Histological findings of pancreatic tissues 72 hrs after MSCs transplantation (H&E; (d) ×200; (e, f) ×400). The yellow arrows showed that in the control group the necrosis and inflammation cells infiltration in pancreas were significantly higher than that in MSCs transplantation group. The blue arrow showed that serious hemorrhage could be found in the control group.

**Figure 4:**
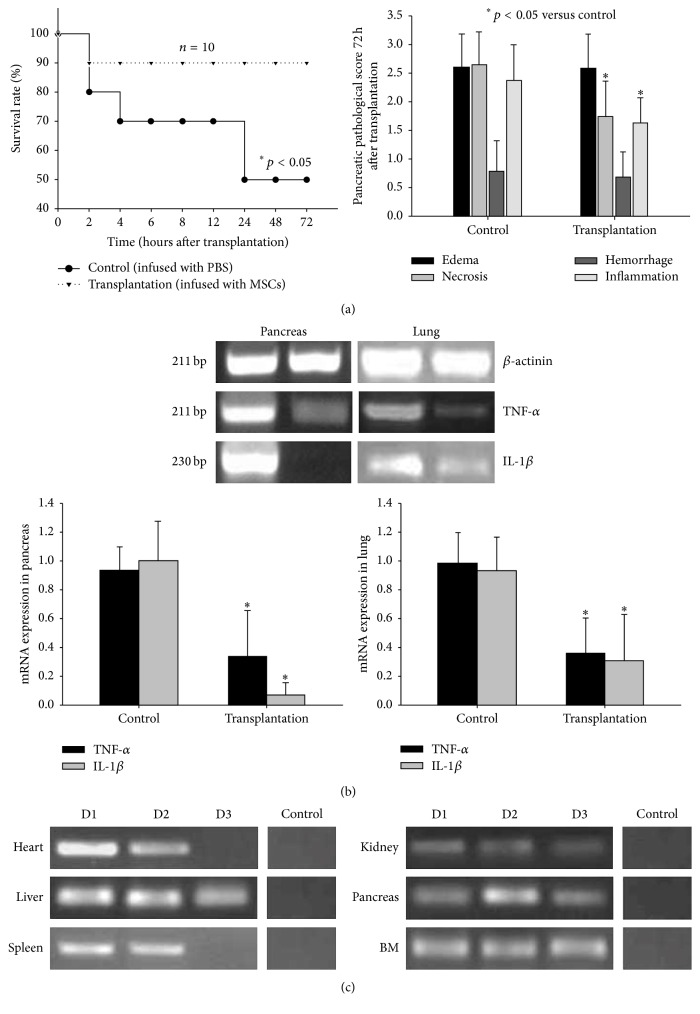
(a) The survival rate of the MSC transplantation group was significantly higher than that of control group (^*∗*^ *p* < 0.05). The survival rate of control group treated with PBS was 50%, while the survival rate of the transplantation group treated with MSCs was 90%. After MSC transplantation, pancreatic pathological scores of necrosis and inflammation decreased significantly (^*∗*^ *p* < 0.05). (b) Detection of expression of TNF-*α* and IL-1*β* mRNA in the pancreas and lungs of control and transplantation groups 72 h after MSC infusion by semiquantitative and real-time RT-PCR. The expression of TNF-*α* and IL-1*β* in the pancreas and lungs was significantly lower in MSC transplantation group than in control group (^*∗*^ *p* < 0.05). (c) Detection of SRY fragment 3 days after MSC transplantation in multiple organs.

After MSC transplantation, the pancreatic necrosis and inflammation were significantly lessened in the transplantation group than in the control group (Figures 3 and 4(a)). The survival rate of the transplantation group was significantly higher compared to the control group (Figure 4(a), *p* < 0.05).

To elucidate the role of MSCs in the therapeutic effects of SAP, expression of two important proinflammatory cytokines TNF-*α* and IFN-*γ* was detected. The relative gene expression of TNF-*α* in the pancreas was 0.933 ± 0.163 in the control group and 0.341 ± 0.316 in the transplantation group. The expression of IL-1*β* in the pancreas was 1.0 ± 0.276 in the control group and 0.073 ± 0.084 in the transplantation group. The expression of TNF-*α* in the lungs was 0.983 ± 0.214 in the control group and 0.360 ± 0.244 in the transplantation group. The expression of IL-1*β* in the lungs was 0.933 ± 0.234 in the control group and 0.311 ± 0.316 in the transplantation group. The expression of TNF-*α* and IL-1*β* mRNA in the transplantation group was significantly lower than that in the control group, also in the pancreas and lungs (Figure 4(b), *p* < 0.05).

To investigate whether MSC could migrate to the target organs, the SRY gene was detected in the heart, liver, spleen, kidney, pancreas, and BM tissue within 2 days after MSC transplantation by using PCR (Figure 4(c)). On the third day, no SRY gene was detected in the heart or spleen (Figure 4(c)). Further study needs to be performed in the future to determine whether there is direct relation between MSC engraftment and therapy effect on SAP and also whether immunomodulatory functions of MSC are involved in it.

## 4. Discussion

Acute pancreatitis is an acute inflammatory process of the pancreas with varying involvement of other regional tissues or remote organ systems. Approximately one-fourth to one-third of patients with severe pancreatitis died from their disease, with a total mortality rate of 2–10% [34]. In 1998, there were 2834 deaths in the United States from acute pancreatitis, making this condition the 14th most common cause of death due to gastrointestinal disease [35]. As SAP is a life-threatening disease, generous efforts have been made to treat SAP more effectively. However, until now, based on the mortality rate of the disease, the prognosis of SAP has not been optimistic.

As pluripotent stem cells, MSCs can differentiate into many cell types* in vitro*. The differentiation ability of tissue-specific stem cells was once thought to be confined to the tissue of origin; however, recent studies have suggested that these cells can also differentiate into other lineages. Jiang et al. have shown that BM MSCs can convert into endothelium, ectoderm, and endoderm at the single-cell level [17]. Using insulin and C-peptide staining, researchers have shown that MSCs can differentiate into insulin-producing cells.* In vivo* functional studies have revealed that the coculture of islets with MSCs provides higher differentiation efficiency [36]. In the current study, we investigated the effect of MSCs on SAP and discussed the feasibility of MSC therapy for SAP.

Injection of taurocholic acid has been used to induce SAP model. The advantage of this method is that the pathogenic mechanism of this experimental SAP model is similar to that of acute biliary pancreatitis [29]. However, the difficulties of the surgery on small animals hindered its application, so cerulein and L-arginine were used to induce SAP in some studies. Because cerulein and L-arginine have different pathogenic mechanisms [37], we think taurocholic acid model is more suitable, and pancreatic necrosis induced by taurocholic is more serious than that in other SAP models. Despite the difficulties of the surgery on small animals, we succeeded in the operation and got stable results. We have evaluated the severity of this model at different time points. The mortality rate in our experimental SAP model was approximately 50% and the severity of disease reached a peak in the first 24 hrs. The regeneration of necrotic tissues in the pancreata of SAP rats was not completed in 7 days.

Regarding the possible protective role that MSCs play in SAP, we engrafted homogenic MSCs to treat experimental SAP rats. Previously other studies have conflicting results about the migration abilities of transplanted MSCs [37]. Some researchers suggested that MSCs migrate to injured pancreas, but liver and other organs were not mentioned [27]. The migration of the MSCs to the pancreas and other organs was proven by PCR of the SRY. Firstly we detected SRY in bone marrow. Migration of MSCs to bone marrow can be called homing. The homing capacity of MSCs is related to expression of Cxcr4. The level of stromal-derived factor 1 in bone marrow is another important factor [38]. MSCs are thought to migrate via the bloodstream to seed new sites of hematopoiesis and to seed various tissues during embryonic and fetal development. The target migration of MSCs to injured tissue has been demonstrated both in human beings and in several experimental animal models [39]. The capacity of MSCs differentiation into pancreatic exocrine cells has not been demonstrated. In the present study whether MSCs can differentiate into pancreatic exocrine cells cannot also be concluded. Previous studies investigated the ability of MSCs to engraft in the pancreas. Certain evidence suggests the possibility that MSCs differentiate into islet *β* cells [40, 41]. Human BM-derived MSCs were applied in experimental acute pancreatitis in rats in a previous study. In that study, infused MSCs were detected in damaged areas of the pancreas [27]. In contrast, in our study, donated MSCs were also found in the lungs and BM, and we evaluated the proliferation ability of MSCs under SAP conditions (results not shown). Migration and adherence of MSCs in the lungs demonstrate protective effects on pancreatitis-associated lung injury [42]. Along with the migration of MSCs, the severity of SAP was reduced in the transplantation group. Indications of reduced SAP were reflected both in levels of tissue inflammatory cytokines and in the survival rate of the animals. The MSCs exhibited valid therapeutic effects on SAP.

The mechanisms regulating MSC migration to peripheral tissues and differentiation into tissue cells are still unknown. The modulation of this homing capacity may be instrumental for harnessing the therapeutic potential of MSCs. Some researchers have shown that CXCR4 overexpression improves MSCs repair ability and that is related to increased release of cytokines and enhanced homing [43]. Recent studies both* in vitro* and* in vivo* have demonstrated that the fusion of MSCs and tissue-specific cells could be one mechanism through which BM-derived nonhematopoietic cells arise [22, 36]. Cells generated* in vivo* through this mechanism form polyploid cells called heterokaryons, which may subsequently give rise to two euploid cells by cytoreductive division. Several reports support the absence or the rare contribution of cell fusion* in vivo*. A recent report evaluated cell fusion events* in vivo* using the Cre/lox system and suggested that the epithelial cells of the lung, liver, and skin can develop from BM-derived cells without cell fusion [44]. In our study, it was unclear whether migrated MSCs differentiated into pancreatic exocrine cells. The therapeutic effects of MSCs on SAP may not exclude the immunoregulatory function of MSCs, which may lessen SIRS. MSCs can alter the outcome of the immune cell response by inhibiting two of the most important proinflammatory cytokines (i.e., TNF-*α* and IFN-*γ*) and increasing the expression of suppressive cytokines, including IL-10. MSCs inhibit the upregulation of several of DC maturation markers, resulting in a decreased capacity to activate alloreactive T cells [26]. Because we demonstrated that IL-1*β* and TNF-*α* mRNA expression was reduced after MSC transplantation in both the lung and the pancreas and that pancreatic pathological scores of necrosis and inflammation decreased, the abatement of local inflammation may play a role in the therapeutic effects. To determine whether MSCs participate in the reconstruction of pancreatic acini as donor cells, further investigation is needed, including colocalization studies of transplanted MSCs with pancreatic cell markers and particular signaling pathway members that have a close relationship with pancreatic development.

## 5. Conclusions

In our study, we have demonstrated that MSC transplantation could improve the prognosis of SAP rats. Engrafted MSCs have homing, migration, and planting capacity during the treatment of SAP. Downregulation of IL-1*β* and TNF-*α* mRNA expression in both the lung and the pancreas was correlated with decrease of pancreatic pathological scores of necrosis and inflammation. Further investigation is needed to determine whether MSCs participate in the reconstruction of pancreatic acini as donor cells.

## Figures and Tables

**Table 1:** The level of serum amylase, AAT, serum creatinine, and pathologic scores in SAP group and control group ($\overset{-}{x}\pm s$).

Item	SAP group (time) (*n* = 5 in each time group)						Control group (*n* = 5)
	0.5 h	2 h	4 h	8 h	12 h	24 h	
Serum amylase (U/L)	4472 ± 865.9^*∗*^	3940 ± 821.3^*∗*^	4584 ± 1844.4^*∗*^	5684 ± 451.5^*∗*^	6721 ± 1154.2^*∗*^	9981 ± 2328.3^*∗∗*^	1431 ± 368.5
AAT (U/L)	35.8 ± 12.68	34.4 ± 6.54	38 ± 13.10	161.6 ± 215.0^*∗∗*^	90.4 ± 30.39	128.6 ± 44.21	31.6 ± 7.92
Serum creatinine (*μ*mol/L)	29.4 ± 9.66	38.2 ± 10.03	32 ± 14.61	38.2 ± 13.16	34.2 ± 12.99	51.4 ± 34.06^*∗∗*^	27.8 ± 8.53
Pathologic scores	2.74 ± 0.27^*∗*^	2.64 ± 0.42^*∗*^	4.58 ± 0.32^*∗*^	6.24 ± 0.36^*∗*^	6.88 ± 0.65^*∗*^	8.41 ± 0.91^*∗∗*^	0.15 ± 0.12

^*∗*^Significantly higher than control group.

^*∗∗*^Significantly higher than all the other groups.
